# Interpretable machine learning in predicting drug-induced liver injury among tuberculosis patients: model development and validation study

**DOI:** 10.1186/s12874-024-02214-5

**Published:** 2024-04-20

**Authors:** Yue Xiao, Yanfei Chen, Ruijian Huang, Feng Jiang, Jifang Zhou, Tianchi Yang

**Affiliations:** 1 School of International Pharmaceutical Business, China Pharmaceutical University, Nanjing, Jiangsu China; 2 Institute of Tuberculosis Prevention and Control, Ningbo Municipal Center for Disease Control and Prevention, No.237, Yongfeng Road, Ningbo, Zhejiang China

**Keywords:** Machine learning, Logistic regression, Tuberculosis, Drug-induced liver injury, Retrospective study

## Abstract

**Background:**

The objective of this research was to create and validate an interpretable prediction model for drug-induced liver injury (DILI) during tuberculosis (TB) treatment.

**Methods:**

A dataset of TB patients from Ningbo City was used to develop models employing the eXtreme Gradient Boosting (XGBoost), random forest (RF), and the least absolute shrinkage and selection operator (LASSO) logistic algorithms. The model's performance was evaluated through various metrics, including the area under the receiver operating characteristic curve (AUROC) and the area under the precision recall curve (AUPR) alongside the decision curve. The Shapley Additive exPlanations (SHAP) method was used to interpret the variable contributions of the superior model.

**Results:**

A total of 7,071 TB patients were identified from the regional healthcare dataset. The study cohort consisted of individuals with a median age of 47 years, 68.0% of whom were male, and 16.3% developed DILI. We utilized part of the high dimensional propensity score (HDPS) method to identify relevant variables and obtained a total of 424 variables. From these, 37 variables were selected for inclusion in a logistic model using LASSO. The dataset was then split into training and validation sets according to a 7:3 ratio. In the validation dataset, the XGBoost model displayed improved overall performance, with an AUROC of 0.89, an AUPR of 0.75, an F1 score of 0.57, and a Brier score of 0.07. Both SHAP analysis and XGBoost model highlighted the contribution of baseline liver-related ailments such as DILI, drug-induced hepatitis (DIH), and fatty liver disease (FLD). Age, alanine transaminase (ALT), and total bilirubin (Tbil) were also linked to DILI status.

**Conclusion:**

XGBoost demonstrates improved predictive performance compared to RF and LASSO logistic in this study. Moreover, the introduction of the SHAP method enhances the clinical understanding and potential application of the model. For further research, external validation and more detailed feature integration are necessary.

**Supplementary Information:**

The online version contains supplementary material available at 10.1186/s12874-024-02214-5.

## Background

Drug-induced liver injury (DILI) presents significant challenges in the context of tuberculosis (TB) treatment. Anti-TB drugs exhibit noteworthy involvement in the occurrence of DILI [1, 2], and the lack of certain early-detection biomarkers [3] further poses challenges to the timely diagnosis and management of DILI. This absence of early detection may result in treatment interruptions and failures amongst TB patients [4, 5], impeding global TB eradication efforts [6]. In China, the elevated incidence rates of DILI in comparison to western nations highlight the potential involvement of traditional Chinese medicines (TCM) and herbal medicines in the development of DILI [7]. This requires addressing various challenges and complexities associated with DILI assessment in a comprehensive and objective manner. Therefore, the primary objective of this study is to develop an optimal predictive model for assessing DILI status, with a specific focus on TB patients within the Chinese context.

The emergence of machine learning (ML) algorithms presents an exciting opportunity to enhance DILI prediction models [8]. Among these, eXtreme Gradient Boosting (XGBoost) [9] and random forest (RF) [10] stand out as two widely-used ensemble learning techniques, each distinguished by its algorithmic approach and features. Selecting the most suitable option between them hinges on the particular characteristics of the data and the prediction objective. Therefore, it is often advisable to conduct experiments with both models to compare their performance.

Nevertheless, one of the primary challenges in implementing ML algorithms in clinical settings is interpreting the outcomes of the models [11, 12]. The Shapley Additive exPlanations (SHAP) framework [13] provides insights into the influence of various features on model predictions and the effect of these features on the DILI status in individuals, thus bridging the interpretability gap.

This study focuses on the development and validation of a prediction model for DILI in the context of TB treatment by using advanced ML algorithms with SHAP interpretability. Through this endeavor, we aim to achieve a balance between accurate prediction and the interpretability of the model, which is crucial for its clinical application.

## Methods

### Data source

The study participants comprised individuals diagnosed with TB at specified hospitals in Ningbo from 1st January 2015 to 2nd January 2020, initially referred by the Chinese Center for Disease Control and Prevention (CDC) [14]. Thereafter, they were connected to administrative records obtained from the electronic health records (EHR) system employed by the local government [15]. The merged dataset comprised demographic information, hospitalization records (both inpatient and outpatient), laboratory tests, and medication profiles.

### Exclusion criteria

**Fig. 1:**
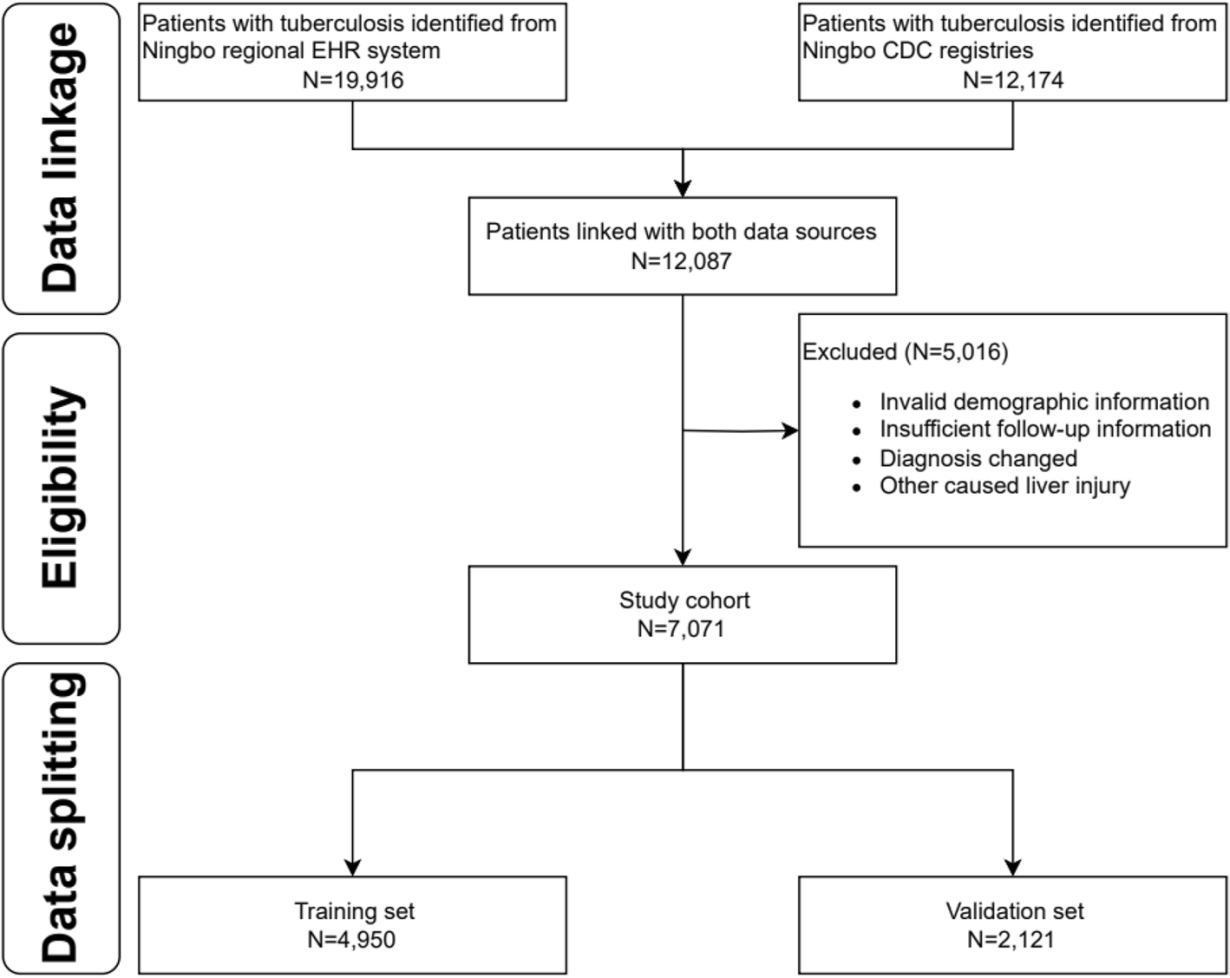
Study schema for subject selection. Abbreviations: EHR, Electronic healthcare record; CDC, Center for Disease Control and Prevention

To ensure consistency in the identification of covariates, individuals with only one health care encounter during the study period were excluded. Furthermore, individuals without ethnicity information and those under 18 years old at diagnosis were not included in the study. The exclusion criteria also filtered out misdiagnosed cases of DILI and liver injuries attributed to known factors like alcohol-related liver disease, non-alcoholic fatty liver disease (NAFLD), and viral hepatitis unrelated to drug-induced causes. The detailed flowchart is presented in Fig. 1.

### Baseline laboratory result collection

For patients included in the study, we defined the baseline period for collecting laboratory test results as from January 1, 2015, to the day before the index diagnosis of pulmonary tuberculosis, as shown in Supplemental Fig. 1. Additionally, liver function test indicators such as alanine transaminase (ALT) or alkaline phosphatase (ALP) were simultaneously examined.

To address the issue of varied baseline definitions in laboratory testing, we utilized two main strategies. Firstly, we employed a binary variable approach to categorize laboratory testing indicators as abnormal or normal, by comparing their values with predefined normal ranges. Secondly, we utilized ratio-based representation to quantify indicator abnormalities, such as calculating ALT multiples relative to the upper limit of the normal (ULN) range.

### Factor identification

In our research, we followed the initial steps outlined in the high dimensional propensity score (HDPS) methodology by Schneeweiss et al. [16]. First, we identified 24 common factors, such as age and gender, to integrate into our models. We then categorized our data into four dimensions: outpatient records, inpatient records, laboratory test records, and medication records. Following the approach of Chen et al. [17], we identified the top 500 most prevalent codes within each dimension. Next, we evaluated code recurrence, classifying codes into three binary variables based on their frequency of occurrence over a 12-month baseline period. This yielded a total of 4*500*3 binary factors. Using a multiplicative model considering binary factor and DILI status, we prioritized covariates and selected the top 400 for inclusion in our final model based on an arbitrary cutoff recommendation [18, 19]. Finally, considering the previously specified 24 variables, our model training ultimately involved incorporating a total of 424 factors.

### DILI diagnostic process

The determination of DILI outcomes followed the revised criteria set forth by the Chinese Society of Hepatology (CSH) DILI consensus, as outlined in Supplemental Table 1 [20].

### Extraction of features used in prediction model

The LASSO regression method, aimed at reducing the number of variables and preventing overfitting [21], was applied to extract significant features for constructing the logistic model. Additionally, both the XGBoost and RF algorithms come equipped with their own feature selection techniques tailored to enhance their respective models.

### Statistical analysis

The study reported the features of both the non-DILI and DILI groups by mean and standard deviation (SD) or as numbers and percentages whenever necessary. Laboratory variables were represented in median and quartiles [22]. The Kruskal–Wallis rank sum test was used for continuous variables, while the chi-square test was used for categorical variables. These analyses were conducted using the statistical software packages SAS 9.4 and R 4.0.3. A statistically significant result was determined with a two-sided *P*-value below 0.05.

### Data splitting

In order to create training and validation sets, a stratified random function in R randomly assigned records at a 7:3 ratio, following conventional practices.

### Parameter optimization

To optimize the parameters of the XGBoost and RF models, a ten-fold cross-validation process combined with grid search [23] was employed. This approach entailed identifying the hyperparameter set that yielded the maximum receiver operating characteristic (ROC). A detailed breakdown of the grid search particulars and optimal results can be found in Supplemental Table 2.

### Model evaluation and interpretation

To assess the model's capacity to differentiate between positive and negative cases, we computed both the area under the receiver operating characteristic curve (AUROC) and the area under the precision recall curve (AUPR) [24]. Calibration was examined through reliability diagrams and Brier scores. Furthermore, the model's clinical utility was evaluated using decision curve analysis. The SHAP technique was utilized to delve deeper into variable contributions. A comprehensive overview of the workflow can be found in Supplemental Fig. 2.

## Results

### Participant and factor identification

The preliminary linkage of data yielded 12,087 instances. Following the application of exclusion criteria, a total of 7,071 subjects were identified as suitable for inclusion in the study.

During a one-year baseline period, we identified the 500 most prevalent codes across each data dimension (outpatient, inpatient, medication, and laboratory test) using the International Classification of Diseases-Tenth Revision (ICD-10), Current Procedural Terminology (CPT), and generic drug names. These items were then categorized into three binary variables: "ever occurring", "sporadically occurring", and "frequently occurring", indicating their recurrence. This process resulted in a total of 6,000 variables, from which the top 400 binary empirical variables were chosen based on their highest risk ratios associated with DILI status. Additionally, the final model incorporated 24 predefined baseline variables, such as gender, age, education level, medication, and maximum ratio of ULN for ALT, ALP, and Tbil, etc. Out of an initial pool of 424 features, 37 were selected for logistic model development using LASSO. The factors included in the LASSO logistic model are detailed in Supplemental Table 3.

### Epidemiology of DILI

The incidence of DILI was observed to be 16.3% overall, with a slightly higher observed in female patients (17.3% *vs.* 15.8%, *p* = 0.134). Detailed demographics and clinical information are outlined in Table 1. Compared to non-DILI individuals, those with DILI demonstrated lower educational attainment and a higher incidence of abnormal baseline levels in ALT and ALP [ALT: 91 (7.9%) *vs.* 273 (4.6%), *p* < 0.001; ALP: 100 (8.7%) *vs.* 400 (6.8%), *p* = 0.023]. Individuals of middle age, females, and those with pre-existing chronic liver conditions were found to have a higher susceptibility to DILI. Significant associations with DILI were identified for certain drugs, including pyrazinamide (PZA), isoniazid (INH), traditional Chinese medicines (TCM), and hepatoprotective agents such as silymarin and glycyrrhetinic acid.

**Table 1:** Demographic and clinical characteristics of patients with and without DILI

	**None-DILI** (*N* = 5,920)		**DILI** (*n* = 1,151)		*P* value	**SMD**
	n	(%)	n	(%)		
**Age at TB diagnosis, years**						
Mean (SD)	47	(19)	48	(18)	0.695	0.013
< 30	1407	(23.8)	243	(21.1)	0.001	0.167
30–49	1843	(31.1)	359	(31.2)		
50–69	1773	(29.9)	414	(35.9)		
70 +	897	(15.1)	135	(11.8)		
**Gender**					0.105	0.053
Male	4051	(68.4)	759	(65.9)		
Female	1869	(31.6)	392	(34.1)		
**Race**					0.668	0.018
Han	5792	(97.8)	1129	(98.1)		
Others	128	(2.2)	22	(1.9)		
**Treatment status**					0.472	0.025
Initial	5464	(92.3)	1070	(93.0)		
Retreatment	456	(7.7)	81	(7.0)		
**Diagnosis site**						0.042
Lung	5509	(93.1)	1083	(94.1)		
Other sites	411	(6.9)	68	(5.9)		
**Education**					0.006	0.112
Illiteracy	2248	(38.0)	488	(42.4)		
Semi-literate	212	(3.6)	37	(3.2)		
Undergraduate	3289	(55.6)	582	(50.6)		
Bachelor	171	(2.9)	44	(3.8)		
**Profession**					0.381	0.072
Industry	3281	(55.4)	625	(54.3)		
Clothing	1718	(29.0)	329	(28.6)		
Retirees	416	(7.0)	85	(7.4)		
Government	235	(4.0)	57	(5.0)		
Others	139	(2.3)	22	(1.9)		
Student	131	(2.2)	33	(2.9)		
**Ratio of ULN of serological markers**^**a**^** Median (IQR)**						
ALT	0.44	(0.27,0.66)	0.52	(0.30,0.66)	< 0.001	0.104
ALP	0.68	(0.56,0.82)	0.68	(0.55,0.82)	0.871	0.047
Tbil	0.52	(0.38,0.71)	0.54	(0.40,0.74)	0.005	0.072
**Serological markers status**						
Abnormal ALT	273	(4.6)	91	(7.9)	< 0.001	0.136
Abnormal ALP	400	(6.8)	100	(8.7)	0.023	0.072
Abnormal Tbil	366	(6.2)	87	(7.6)	0.093	0.054
**Comorbidities**						
Diabetes	599	(10.1)	102	(8.9)	0.211	0.043
Hypertension	1108	18.7	216	18.8	1.000	0.001
Liver-related diseases^b^	624	10.5	224	19.5	< 0.001	0.252
HBV	105	1.8	17	1.5	0.560	0.023
**Medication**						
TCM	1250	(21.1)	323	(28.1)	< 0.001	0.162
Hepatoprotective agents^c^	1971	(33.3)	486	(42.2)	< 0.001	0.192
PZA	1105	(18.7)	304	(26.4)	< 0.001	0.186
RIF	590	(10.0)	121	(10.5)	0.610	0.018
INH	1000	(16.9)	267	(23.2)	< 0.001	0.158

*Abbreviations*: *ALP* Alkaline phosphatase, *ALT* Alanine aminotransferase, *DILI* Drug-induced liver injury, *Tbil* Total serum bilirubin, *ULN* Upper limit of normal, *HBV* hepatitis B virus, *TB* tuberculosis, *TCM* Traditional Chinese medicine, *PZA* Pyrazinamide, *RIF* Rifampicin, *INH* Isoniazid, *SMD* Standardized mean difference

^a^Continuous variables

^b^Liver-related diseases included liver insufficiency, abnormal liver function, hepatitis, fatty liver, alcoholic liver, jaundice

^c^Hepatoprotective agents included silymarin, glycyrrhetinic acid and others

### Model development and validation

**Fig. 2:**
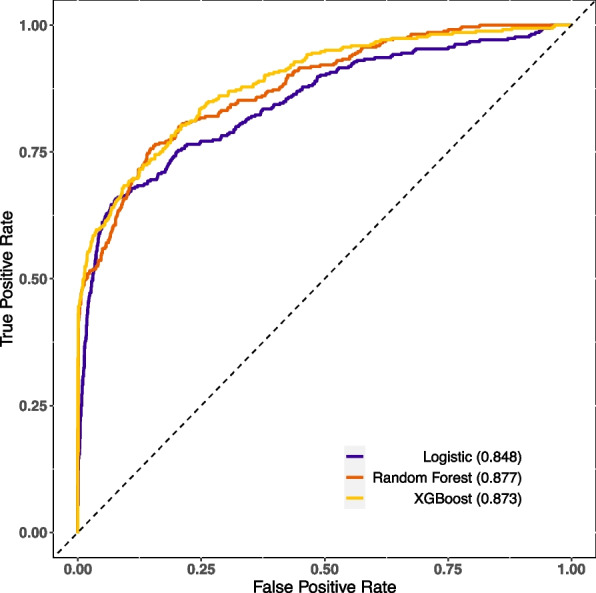
Comparison of the AUROC of the XGBoost, logistic and random forest in the validation set

**Fig. 3:**
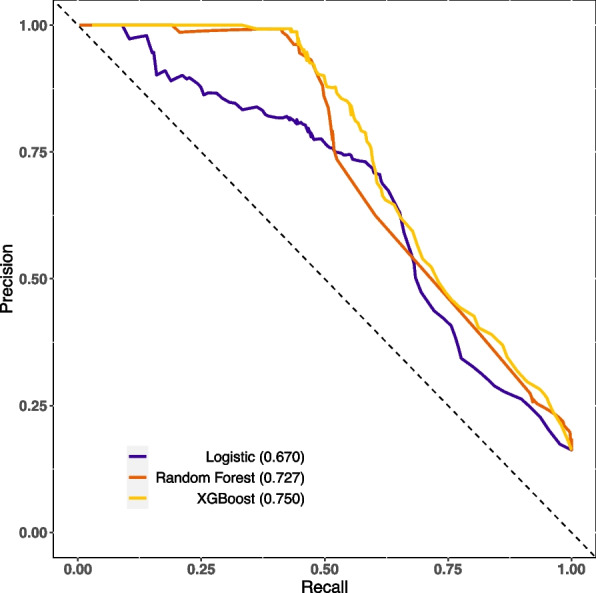
Comparison of the AUPR of the XGBoost, logistic and random forest in the validation set

**Fig. 4:**
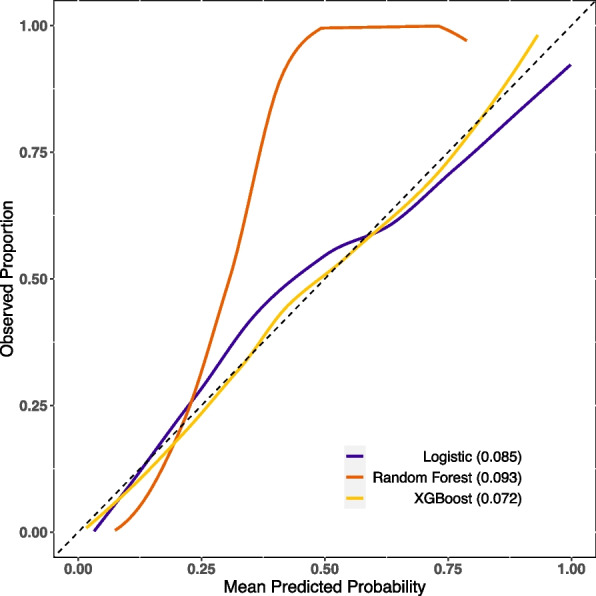
Comparison of the calibration curve of the XGBoost, logistic and random forest in the validation set

**Fig. 5:**
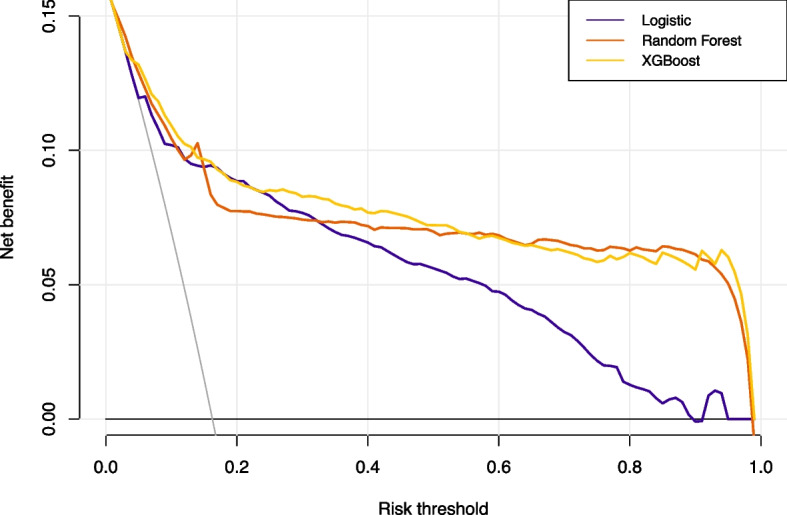
The decision curve of the XGBoost, logistic and random forest in the validation set

The XGBoost and RF models were constructed using optimal parameters obtained through the previously mentioned GridSearchCV method. The LASSO logistic model was constructed with the aforementioned variables. Internal validation was conducted by partitioning validation sets, resulting in a comparison of model performance among the three models showcased in Table 2. The XGBoost model exhibited slightly superior discriminatory ability when compared with the RF and LASSO logistic model, with AUROC values of 0.89 versus 0.88/0.85 and AUPR values of 0.75 versus 0.73/0.67, respectively, as shown in Figs. 2 and 3. The RF model demonstrated increased recall with a score of 0.78, while the XGBoost model achieved the highest F1-score of 0.57. Calibration was evaluated through ten predictive probability-based bins and verified by the reliability diagram presented in Fig. 4, supported by a Brier score of 0.08, indicating the impressive alignment in calibration between the XGBoost and LASSO logistic models. Extensive analysis of the decision curve revealed positive net benefits for all models. Notably, XGBoost models outperformed both the RF and LASSO logistic models within the threshold range of approximately 0.2 to 0.5, as demonstrated in Fig. 5.

**Table 2:** Comparison of performance ability of the three models in the validation set

Performance ability	Indicators	Logistic	Random Forest	XGBoost
Discrimination	Optimal cutoff (Youden)	0.144	0.182	0.167
	True negative	1332	1424	1461
	False positive	444	352	315
	False negative	79	75	83
	True positive	266	270	262
	Specificity	0.750	0.802	0.823
	Sensitivity	0.771	0.782	0.760
	Precision	0.375	0.434	0.454
	Recall	0.771	0.782	0.760
	F1 score	0.505	0.558	0.568
	Accuracy	0.753	0.799	0.812
	AUROC	0.848	0.877	0.887
	AUPR	0.670	0.727	0.750
Calibration	Brier score	0.085	0.093	0.072

*Abbreviations AUROC* Area under receiver operative curve, *AUPR* Area under the precision-recall curve

### Model interpretation

**Fig. 6:**
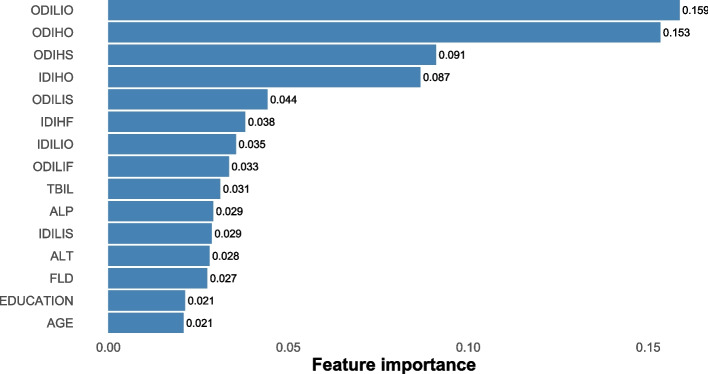
Top important features selected by XGBoost (> 0.01). Abbreviations: ODILIO, outpatient drug-induced liver injury, once occurring; ODIHO, outpatient drug induced hepatitis, once occurring; ODIHS, outpatient drug induced hepatitis, sporadically occurring; IDIHO, inpatient drug induced hepatitis, once occurring; ODILIS, outpatient drug induced liver injury, sporadically occurring; IDIHF, inpatient drug induced hepatitis, frequently occurring; IDILIO, inpatient drug induced liver injury, once occurring; ODILIF, outpatient drug induced liver injury, frequently occurring; TBIL, total bilirubin; ALP, alkaline phosphatase; IDILIS, inpatient drug induced liver injury, sporadically occurring; ALT, alanine aminotransferase; FLD, fatty liver disease

Revealing the factors that influenced the outperformed model's predictions, Fig. 6 laid out the most paramount features of XGBoost (with feature importance > 0.01). Of note, historical occurrences of DILI, DIH, and fatty liver disease (FLD) during the baseline phase were consistently highlighted. Moreover, the ULN for ALT, ALP and Tbil were also identified as critical factors. The SHAP values calculated for the XGBoost model, as shown in Supplemental Fig. 3, indicate that individuals who had chronic liver disease during baseline were more likely to be in DILI status. Interestingly, we found that those with a lower educational level were more susceptible to DILI status. To gain a deeper understanding of the underlying mechanism and the effects of features in the XGBoost model, we randomly selected two typical patients from the dataset. Furthermore, we created force plots to visualize their decision process, as illustrated in Supplemental Fig. 4 and Supplemental Fig. 5. The average SHAP value was 0.168, where yellow indicates a positive impact and purple represents a negative impact. In Supplemental Fig. 4, the identified patient with a SHAP value of 1.06, surpassing the average, is likely to develop DILI. The significant influencing factor is being diagnosed with DILI or DIH at least once during the baseline period. The same rationale applies to the identified patient as depicted in Supplemental Fig. 5. Additionally, Supplemental Fig. 6 presents a force plot that captures the aggregate effect in the validation set.

## Discussion

To our knowledge, this study represents the initial attempt to evaluate the prediction for DILI in an Asian population, predominantly of Han ethnicity, with TB using regional electronic health records. We observed slightly enhanced discrimination abilities in ML models compared to the logistic model. While logistic regression offers better clinical generalizability, it struggles with overfitting and handling missing variables, resulting in overall weaker performance than anticipated. In contrast, both XGBoost and RF employ more advanced techniques. XGBoost utilizes gradient boosting, progressively building weak learners and effectively capturing non-linear relationships with built-in regularization. On the other hand, RF, a bagging ensemble method, constructs independent decision trees on random subsets of data, resulting in robust averaging but with less explicit regularization. XGBoost excels in capturing intricate non-linear patterns, making it suitable for tasks involving complex and dynamic interactions like predicting DILI during TB treatment. Its training efficiency is also evident when handling large datasets. RF, with its robust averaging, is well-suited for further application in diverse datasets but may encounter challenges in effectively capturing subtle non-linear patterns among multiple explanatory variables.

Several prior studies have identified risk factors associated with DILI during TB treatment, involving chronic liver disease, specific drug combinations, age, and various demographic characteristics [25–27]. Lammert et al. [28] suggested an increased risk of DILI in patients with chronic liver disease indicative of NAFLD. Chang et al. [29] indicated a significant rise in hepatotoxicity risk associated with adding PZA to INH and RIF. Hosford et al. [30] established a notable elevation in hepatotoxicity risk among individuals over 60 years of age through a systematic literature review. Abbara et al. [2] found low patient weight, HIV-1 co-infection, higher baseline ALP levels, and alcohol intake were risk factors. Thus, in our model, we predefined enzyme levels, utilization of anti-TB drugs such as PZA, INH, and RIF, hepatoprotective agents such as silymarin and glycyrrhetinic acid, alcohol intake, and demographic variables such as age, gender, education level, ethnicity, profession as predictors. In the ultimate XGBoost model, the contribution weights for chronic liver disease, ULN of ALT, ALP, Tbil, and age surpass 0.01, consistent with earlier research discoveries.

Currently, a range of predictive models for DILI primarily operates at the molecular level in preclinical settings [31], utilizing diverse artificial intelligence assisted algorithms [32]. Minerali et al. [33] employed the Bayesian ML method, resulting in an AUROC of 0.81, 74% sensitivity, 76% specificity, and 75% accuracy. Xu et al. [34] proposed a deep learning model, achieving 87% accuracy, 83% sensitivity, 93% specificity, and an AUROC of 0.96. Dominic et al.'s Bayesian prediction model [35] demonstrated balanced performance with 86% accuracy, 87% sensitivity, 85% specificity, 92% positive predictive value, and 78% negative predictive value. In the clinical stage, only Zhong et al. introduced a single tree XGBoost model with 90% precision, 74% recall, and 76% classification accuracy for DILI prediction, using a clinical sample of 743 TB cases [36]. In our study, we leveraged regional healthcare data and employed the XGBoost algorithm. The model exhibited 76% recall, 82% specificity, and 81% accuracy in predicting DILI status. Our approach was proven robust, as evidenced by a mean AUROC of 0.89 and AUPR of 0.75 upon tenfold cross validation. During the clinical treatment stage, our model exhibited high levels of accuracy and interpretability.

The choice of a cutoff in a DILI prediction model is crucial and depends on specific study goals and requirements. Various studies have investigated optimal cutoff values in DILI prediction models to enhance understanding and prediction accuracy. For instance, in a study focused on drug-induced liver tumors, the maximum Youden index was utilized to determine the ideal cutoff point [37]. Another study, aimed at predicting DILI and cardiotoxicity, determined 0.4 as the optimal cutoff value using chemical structure and in vitro assay data [38]. Similarly, a system named DILIps, designed to predict DILI in drug safety, utilized the ROC curve to select the best cutoff value [39]. Given the imbalanced dataset in our study, we found the precision recall curve method seemed to be more appropriate. Additionally, considering the severe consequences of DILI, prioritizing the detection of DILI suggests choosing a lower cutoff to maximize sensitivity. Thus, in our study, we opted for the maximum Youden index as the best cutoff.

However, the acceptability of ML in the medical community faces a significant hurdle regarding interpretability, particularly in settings where clinical decisions are paramount. Our research employed SHAP strategies to illuminate the complex mechanisms of the XGBoost model.

## Strengths and limitations

The study utilized a large dataset of over 7,000 TB patients to develop a robust model and comprehensively included clinical, demographic, and biochemical variables to improve predictive accuracy. Furthermore, the model incorporates SHAP analysis to improve interpretability. However, as we embark on the integration of ML into clinical settings, a vital concern persists regarding the generalizability of models [40]. While our model demonstrates enhanced predictive accuracy, it's important to recognize the inherent limitations stemming from the lack of external validation. Patient characteristics [41] and drug interactions [42] may differ widely across populations. This underscores the importance of validating models on diverse patient cohorts and geographical regions. Moreover, the study's reliance on a data-driven approach and the inherent complexity of integrating ML models into clinical practice present additional limitations [43]. Additionally, the dependence on clinical diagnosis for DILI and the potential influence of unmeasured variables on model accuracy are acknowledged. While the study's findings offer valuable insights, careful consideration is warranted when interpreting them.

## Conclusions

XGBoost shows improved predictive performance compared to RF and LASSO logistics in this study. Moreover, introducing the SHAP method enhances the clinical understanding and potential application of the model. For further research, external validation and more detailed feature integration are necessary.

### Code availability statement

To enhance reproducibility and facilitate peer review, we uploaded the code used for model fitting. The source code associated with this research is available on the GitHub repository (https://github.com/cpu-pharmacoepi/TB-DILI). For inquiries or assistance related to the code, please contact 1,020,202,613@cpu.edu.cn.

### Supplementary Information

**Supplementary Material 1.**

**Supplementary Material 2.**

## Data Availability

The datasets used and analyzed during the current study are available from the corresponding author on reasonable request. Data cannot be shared publicly because of privacy and confidentially of the TB patients in Ningbo, Zhejiang, China.

## Abbreviations

ALP: Alkaline phosphatase
*ALT*: Alanine transaminase
*AUPR*: Area under the precision recall curve
AUROC: Area under the receiver operating characteristic curve
*CDC*: Center for Disease Control and Prevention
CPT: Current procedural terminology
CSH: Chinese Society of Hepatology
DIH: Drug-induced hepatitis
DILI: Drug-induced liver injury
EHR: Electronic healthcare record
EMB: Ethambutol
FLD: Fatty liver disease
HDPS: High dimensional propensity score
ICD-10: International Classification of Diseases-Tenth Revision
INH: Isoniazid
INR: International normalized ratio
LASSO: Least Absolute Shrinkage and Selection Operator
ML: Machine learning
NAFLD: Nonalcoholic fatty liver disease
PZA: Pyrazinamide
RF: Random Forest
RIF: Rifampicin
ROC: Receiver operating characteristic
SD: Standard deviation
SHAP: Shapley Additive exPlanations
SMD: Standardized mean difference
TB: Tuberculosis
Tbil: Total serum bilirubin
TCM: Traditional Chinese medicine
ULN: Upper limit of normal
XGBoost: EXtreme Gradient Boosting
