# Supplementary material for: Interpretable machine learning in predicting drug-induced liver injury among tuberculosis patients: model development and validation study
Source: BMC Med Res Methodol. 2024 Apr 20;24:92. doi: 10.1186/s12874-024-02214-5 (PMC11031978; doi:10.1186/s12874-024-02214-5)
Supplement: Supplementary file 1 — Supplementary Material 1. [file 12874_2024_2214_MOESM1_ESM.docx]

**Supplemental Figure 1**: Study cohort construction





TB: tuberculosis; DILI: drug induced liver injury

**Supplemental Figure 2**: Study design and workflow


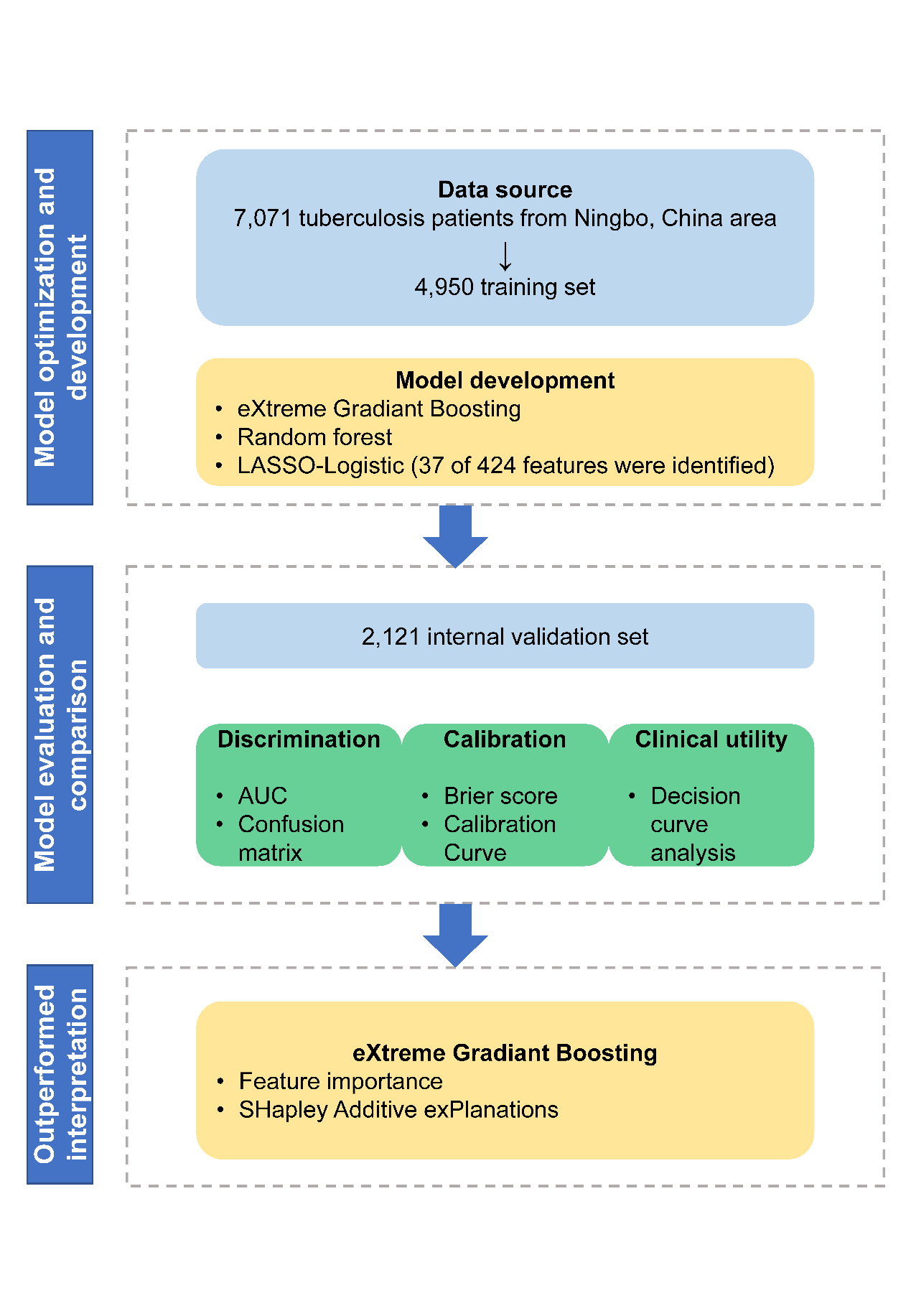


Abbreviation: LASSO, least absolute shrinkage and selection operator; AUC, area under curve.

**Supplemental Figure 3**: SHAP value summary plot of XGBoost prediction model


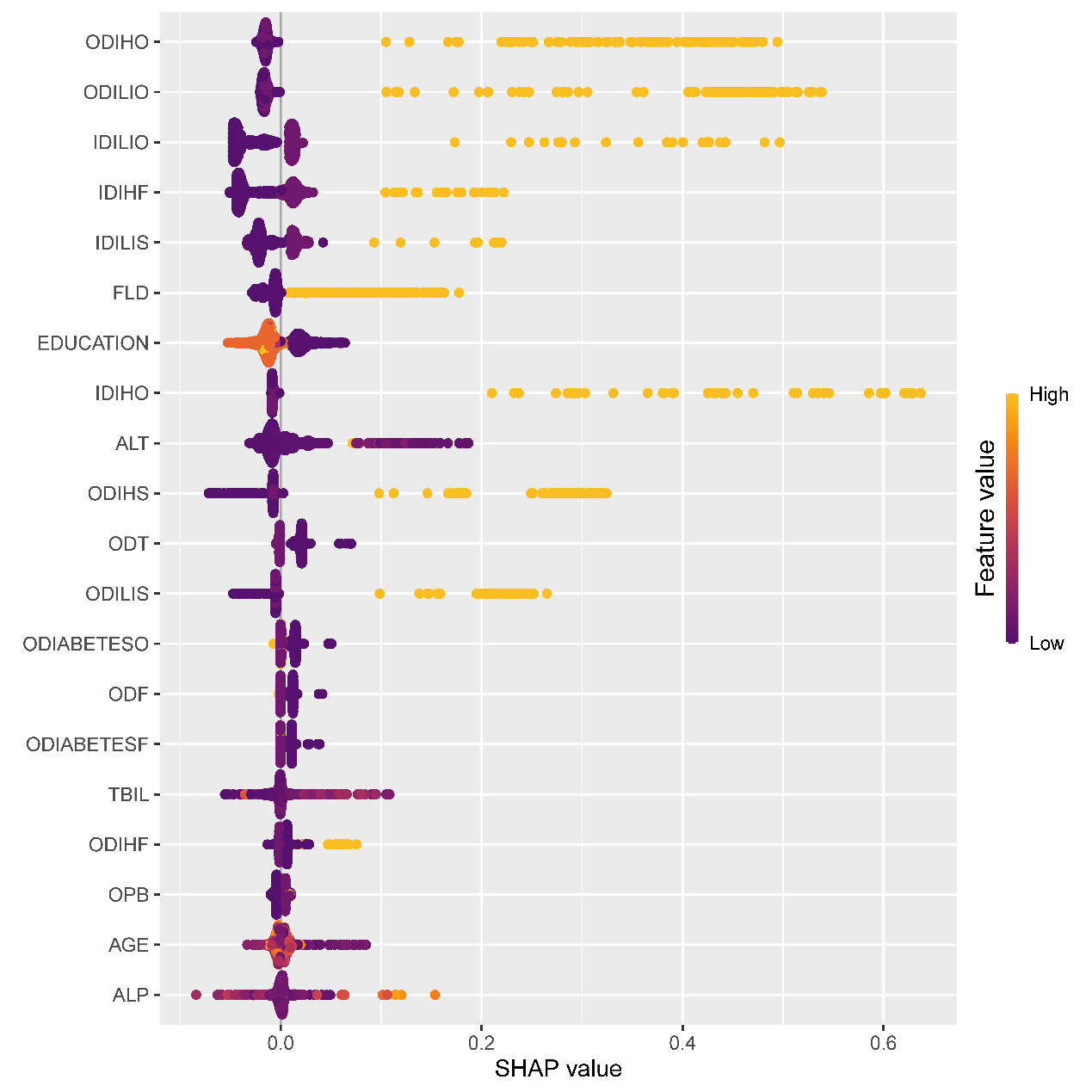


Abbreviation: SHAP, The SHapley Additive exPlanations; XGBoost, eXtreme Gradient Boosting; ODILIO, outpatient drug-induced liver injury, once occurring; ODIHO, outpatient drug induced hepatitis, once occurring; ODIHS, outpatient drug induced hepatitis, sporadically occurring; IDIHO, inpatient drug induced hepatitis, once occurring; ODILIS, outpatient drug induced liver injury, sporadically occurring; IDIHF, inpatient drug induced hepatitis; ODT, outpatient decsyed teeth; ODF, outpatient dermatitis; OPB, outpatient pediatric bronchitis; ODIABETESO, outpatient diabetes, once occurring; ODIABETESP, outpatient diabetes, frequently occurring.

**Supplemental Figure 4**: SHAP for positive individual of XGBoost prediction model


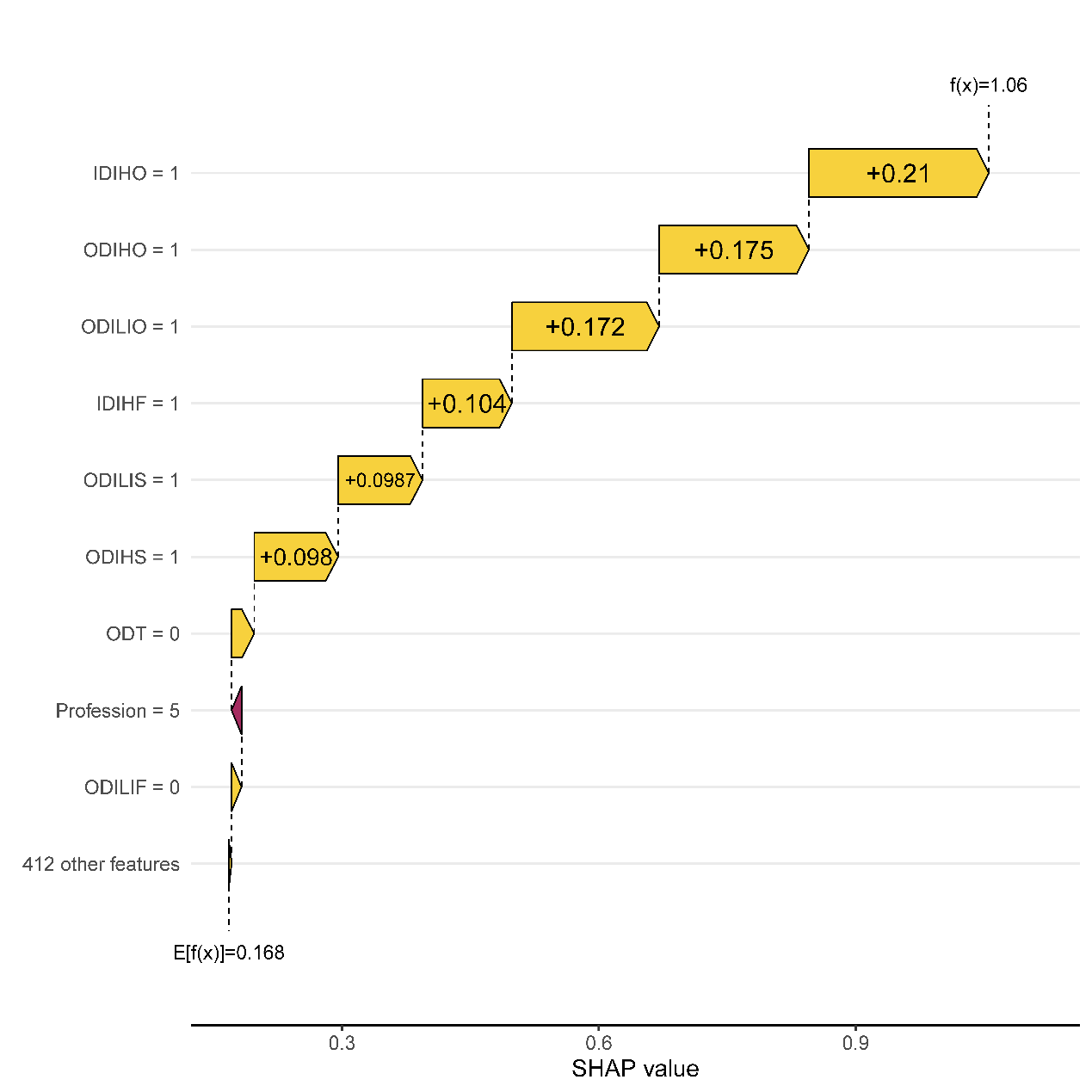


Abbreviation: SHAP, The SHapley Additive exPlanations; XGBoost, eXtreme Gradient Boosting; ODILIO, outpatient drug-induced liver injury, once occurring; ODIHO, outpatient drug induced hepatitis, once occurring; ODIHS, outpatient drug induced hepatitis, sporadically occurring; IDIHO, inpatient drug induced hepatitis, once occurring; ODILIS, outpatient drug induced liver injury, sporadically occurring; IDIHF, inpatient drug induced hepatitis; ODT, outpatient decsyed teeth; ODILIF, outpatient drug induced liver injury, frequently occurring.

**Supplemental Figure 5**: SHAP for negative individual of XGBoost prediction model


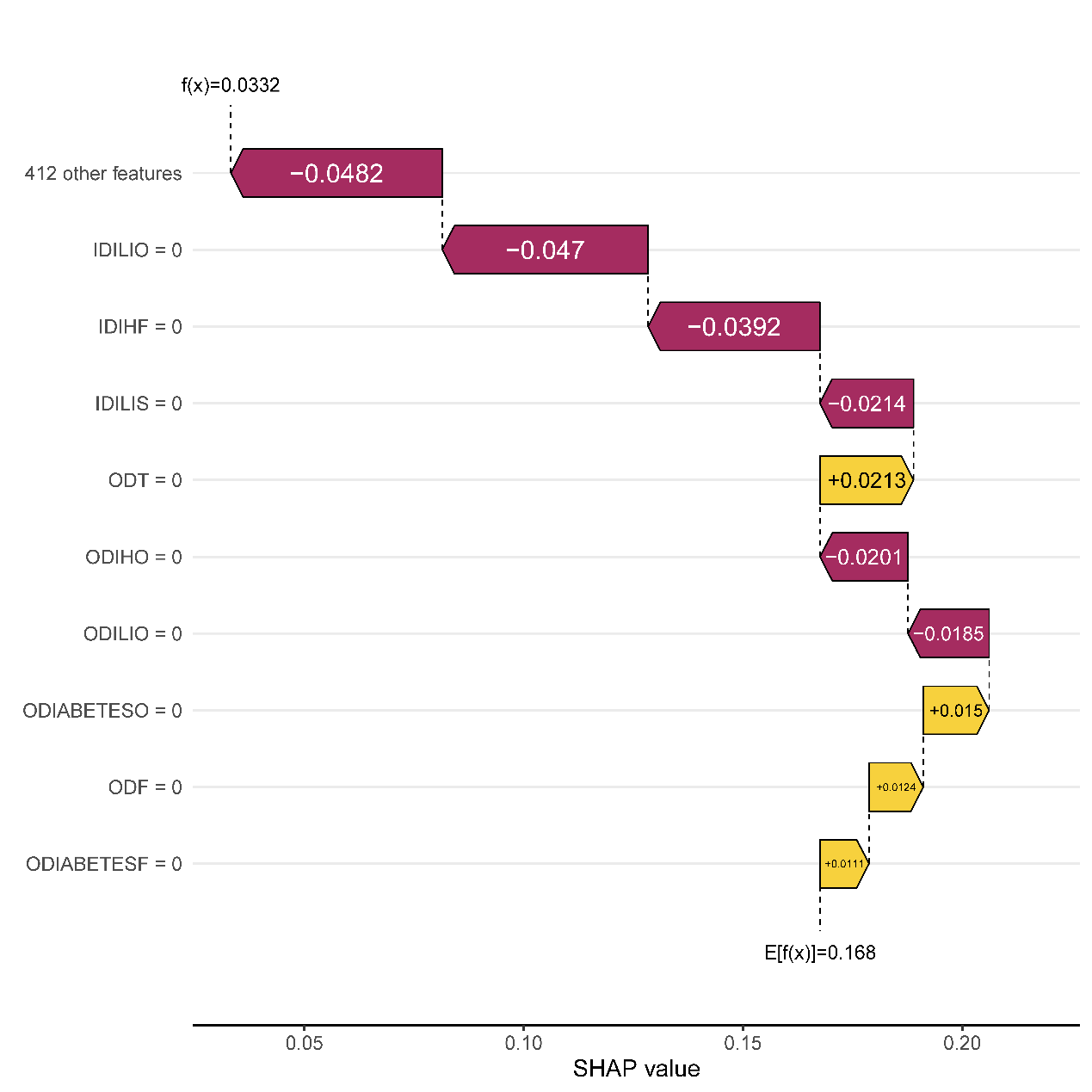


Abbreviation: SHAP, The SHapley Additive exPlanations; XGBoost, eXtreme Gradient Boosting; ODILIO, outpatient drug-induced liver injury, once occurring; IDILIO, inpatient drug-induced liver injury, once occurring; ODIHO, outpatient drug induced hepatitis, once occurring; IDILIS, inpatient drug induced liver injury, sporadically occurring; IDIHF, inpatient drug induced hepatitis; ODT, outpatient decsyed teeth; ODF, outpatient dermatitis; ODIABETESO, outpatient diabetes, once occurring; ODIABETESP, outpatient diabetes, frequently occurring.

**Supplemental Figure 6**: Aggregate SHAP value in validation set of XGBoost prediction model


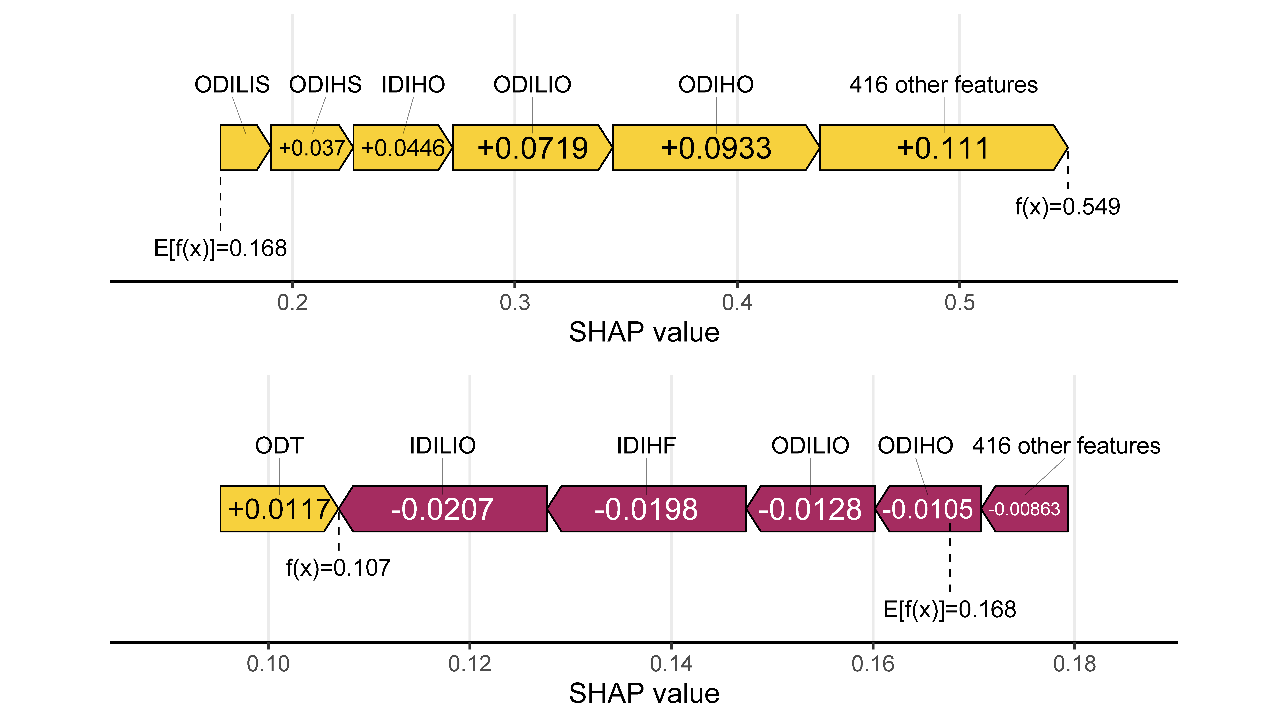


Abbreviation: SHAP, The SHapley Additive exPlanations; XGBoost, eXtreme Gradient Boosting; ODILIO, outpatient drug-induced liver injury, once occurring; IDILIO, inpatient drug-induced liver injury, once occurring; ODIHO, outpatient drug induced hepatitis, once occurring; IDILIS, inpatient drug induced liver injury, sporadically occurring; IDIHF, inpatient drug induced hepatitis; ODT, outpatient decsyed teeth; ODILIS, outpatient drug induced liver injury, sporadically occurring; ODIHS, outpatient drug induced hepatitis, sporadically occurring; IDIHO, inpatient drug induced hepatitis, once occurring.
