# Supplementary material for: Interpretable machine learning in predicting drug-induced liver injury among tuberculosis patients: model development and validation study
Source: BMC Med Res Methodol. 2024 Apr 20;24:92. doi: 10.1186/s12874-024-02214-5 (PMC11031978; doi:10.1186/s12874-024-02214-5)
Supplement: Supplementary file 2 — Supplementary Material 2. [file 12874_2024_2214_MOESM2_ESM.docx]

**Supplemental Table 1:** Definition and criteria for drug-induced liver injury

| **Grade level** | **Definition** | **Description** |
| --- | --- | --- |
| 0 | No liver injury | Patients tolerate drug treatment and have no hepatotoxic reactions. |
| 1 | Mild liver injury | Elevations in serum ALT and/or ALP levels, TBil <2.5 ULN (2.5 mg/dL or 42.75 μmol/L), INR <1.5. Most patients show adaptability to the liver injury. Patients may present with or without symptoms such as fatigue, asthenia, nausea, anorexia, right upper abdominal pain, jaundice, pruritus, rashes, or weight loss. |
| 2 | Moderate liver injury | Elevated serum ALT and/or ALP, with TBil ≥2.5 ULN or INR ≥1.5. The above mentioned symptoms may become aggravated. |
| 3 | Severe liver injury | Elevated serum ALT and/or ALP, TBil ≥5 ULN (5 mg/dL or 85.5 μmol/L) with or without INR ≥1.5. The symptoms are further aggravated, which indicates the need of hospitalization or delayed hospital stay, but there is no evidence of hepatic encephalopathy. |
| 4 | ALF | Evidence of coagulation abnormality indicated by INR ≥1.5 or PTA <40%, signs of hepatic encephalopathy, and TBil ≥10 ULN (10 mg/dL or 171 μmol/L) or daily elevation ≥1.0 mg/dL (17.1 μmol/L) in 26 weeks after the DILI onset. Patients may have ascites and DILI-related dysfunction of other organs. If there is evidence of underlying chronic liver diseases, especially liver cirrhosis, the diagnosis of ACLF is established. |
| 5 | Lethal | Death due to DILI, or need to receive liver transplantation for survival. |

Abbreviation: ALT, Alanine transaminase; ALP, Alkaline phosphatase; TBil, Total bilirubin; INR, International normalized ratio; ALF, Acute liver failure; ULN, upper limit of normal; PTA, Plasma thromboplastin antecedent; DILI, drug-induced liver injury; ACLF, Acute-on-chronic liver failure.

**Supplemental Table 2:** The arbitrary setting and optimal value in XGBoost and RF in grid search method

| **Method** | **Hyperparameter** | **Range** | **Optimal value** |
| --- | --- | --- | --- |
| RF | Randomly selected predictors | 2, 4, 6, 8, 10 | 10 |
|  | Splitting rule | Gini, Extra trees, Hellinger | Hellinger |
|  | Minimal node size | 2, 4, 6, 8, 10 | 6 |
| XGBoost | Boosting iterations | 20, 40, 60 | 60 |
|  | Max tree depth | 2, 6, 10 | 6 |
|  | Shrinkage | 0.1, 0.01, 0.001 | 0.1 |
|  | Subsample ratio of columns | 0.8, 0.9, 1 | 1 |
|  | Subsample percentage | 0.8, 0.9, 1 | 0.9 |

Abbreviation: XGBoost, eXtreme Gradient Boosting; RF, Random Forest.

**Supplemental Table 3:** The details and variance inflation factor of features selected in the final LASSO logistic model

| **Feature source** | **Feature name** | **Abbreviations** | **Feature type** | **CDC National Clinical v2.0** | **Variance inflation factor** |
| --- | --- | --- | --- | --- | --- |
| Outpatient record | Drug-induced hepatitis | ODIH | Binary | K71.601 | 1.07 |
|  | Unspecified liver disease | OULD | Binary | K76.900 | 1.02 |
|  | Hyperlipidemia | OH | Binary | E78.500 | 1.07 |
|  | Abnormal results of liver function tests | OARL | Binary | R94.500 | 1.03 |
|  | Drug-induced liver injury | ODILI | Binary | K71.901 | 1.03 |
|  | stomachache | OS | Binary | R10.400 | 1.02 |
|  | Drug-induced dermatitis | ODID | Binary | L27.005 | 1.01 |
|  | Thyroid nodules | OTN | Binary | E04.101 | 1.01 |
|  | Chronic hepatitis | OCHS/OCHO/OCHF* | Binary | K73.900 | 2.31/2.27/3.53 |
|  | Pathological negative tuberculosis | OPNTB | Binary | A16.020 | 1.04 |
| Inpatient record | Drug-induced liver injury | IDILI | Binary | K71.901 | 1.05 |
|  | 0.9% Sodium chloride injection | IPSCI | Binary | / | 1.08 |
| Baseline record | Sex | Gender | Binary | / | 1.05 |
|  | Race | Race | Binary | / | 1.03 |
|  | Profession | Profession | Categorical | / | 1.02 |
|  | Type of diagnosis | Diagnosis | Binary | / | 1.06 |
|  | Type of tuberculosis | tuberculosis | Binary | / | 1.03 |
|  | Education | Education | Ordinal | / | 1.06 |
|  | Age | Age | Categorical | / | 1.50 |
|  | Diabetes | Diabetes | Binary | / | 1.16 |
|  | Liver-related disease | Liver | Binary | / | 1.35 |
|  | Hypertension | Hypertension | Binary | / | 1.45 |
|  | viral hepatitis type B | HBV | Binary | / | 1.14 |
|  | Alcohol consumption | Drunk | Binary | / | 1.03 |
| Laboratory record | ULN of ALT | ALT_ratio | Numerical | / | 1.56 |
|  | ULN of ALP | ALP_ratio | Numerical | / | 1.93 |
|  | ULN of Tbil | Tbil_ratio | Numerical | / | 1.91 |
|  | Abnormal results of ALT tests | ALT | Binary | / | 1.89 |
|  | Abnormal results of ALP tests | ALP | Binary | / | 1.91 |
|  | Abnormal results of Tbil tests | Tbil | Binary | / | 1.89 |
| Medication record | Traditional Chinese medicine | TCM | Binary | / | 1.17 |
|  | The count of hepatoprotective agents | Hepato | Numerical | / | 1.35 |
|  | Pyrazinamide | Pyrazinamide | Binary | / | 2.12 |
|  | Rifampicin | Rifampicin | Binary | / | 1.52 |
|  | Isoniazid | Isoniazid | Binary | / | 2.24 |

* represents three features due to the high dimensional propensity score matching method.

Abbreviation: ALP, alkaline phosphatase; ALT, alanine aminotransferase; Tbil, total serum bilirubin; ULN,upper limit of normal
